# Striatal Functional Connectivity Alterations After Two-Week Antidepressant Treatment Associated to Enduring Clinical Improvement in Major Depressive Disorder

**DOI:** 10.3389/fpsyt.2019.00884

**Published:** 2019-12-06

**Authors:** Jing An, Le Li, Li Wang, Yun-Ai Su, Ying Wang, Ke Li, Yawei Zeng, Qingmei Kong, Chaogan Yan, Tianmei Si

**Affiliations:** ^1^Clinical Psychopharmacology Division, Peking University Institute of Mental Health (Sixth Hospital) & National Clinical Research Center for Mental Disorders/Key Laboratory of Mental Health, Ministry of Health (Peking University), Beijing, China; ^2^Beijing Suicide Research and Prevention Center, Beijing Huilongguan Hospital, Beijing, China; ^3^CAS Key Laboratory of Behavioral Science, Institute of Psychology, Beijing, China; ^4^The 984th Hospital of People's Liberation Army of China, Beijing, China; ^5^Department of Radiology, 306 Hospital of People's Liberation Army, Beijing, China

**Keywords:** major depressive disorder, antidepressant, striatum, prediction, clinical improvement

## Abstract

**Background:** Only less than 40% of patients with Major depressive disorder (MDD) can achieve remission after several weeks of initial antidepressant treatment. Predicting whether the prescribed treatment is effective in the following course may help clinicians modify the treatment regimen in time, and reduce the staggering burden for patients and society. However, there are not yet reliable markers based on neurobiological change after a treatment regimen steadily applied, for predicting clinical treatment outcome. The striatal circuits often exhibit abnormality for MDD patients, and are implicated in antidepressant treatments.

**Methods:** Nineteen first-episode drug-naive MDD patients (nine females, mean age was 30 years old) were recruited to undergo clinical symptom assessment and resting state fMRI scanning at baseline, after 2 and 8 weeks of treatment with duloxetine. A seed-based analysis was used to obtain functional connectivity (FC) maps of six sub-regions of the stratum, then we explored the relationship of 2-week changes of striatal FC with clinical symptom improvement after 8-week duloxetine treatment.

**Results:** The results revealed that 2-week FC changes of the striatal cognitive and affective subdivisions with the frontoparietal regions positively correlated with 8-week symptom improvement. We also found that early FC changes between the striatal motor subdivision and the motor-related cortical regions negatively correlated with later symptom improvement.

**Conclusions:** These findings suggest that change of the FC of the cortical-striatal circuits at the early stage of treatment is critical for later remission of MDD. Furthermore, the association between the FC change and symptom improvement may have significant implication for clinical practice to regard neural changes as reference for evaluating how antidepressant treatment works.

## Introduction

Major depressive disorder (MDD) is a common and disabling psychiatric disorder, with multi-dimensional clinical symptoms involving emotion, cognition, psychomotor, and somatic domains (1). Though antidepressant medication is used as first-line treatment for MDD, only less than 40% of patients achieve remission with initial treatment (2, 3). As suggested by current treatment guidelines, treatment regimen should be changed if there has not been a partial response after 4 to 6 weeks (1, 4), which make non-respondent patients endure prolonged ineffective treatment and largely increase disease burden. Hence, it is of great value to identify indicators of response to a given antidepressant treatment early in the treatment process. However, the neural mechanism underlying the predictor of treatment outcome is unclear. Several lines of clinical evidence have shown that early improvement of clinical symptoms within the first 2 weeks of antidepressant treatment can partly predict later treatment outcome (5, 6), highlighting 2-week as a key time frame in determining long-term clinical therapeutic effect. In addition, from a clinical perspective, the antidepressant treatment may take 2 weeks to have a stable effect on the brain after dose titration to the target treatment dose or reaching steady state.

Functional magnetic resonance imaging (fMRI) have provided a powerful tool to investigate the changes of brain activity and functional networks of MDD patients during treatment, which may help to identify some reliable and objective predictors of treatment outcome (7). In the domain of neuroimaging, neural systems supporting emotion processing and regulation, and reward seeking are found dysfunctional in MDD, which mainly can be conceptualized as emotion network and reward network (8). Reward network focusing on ventral striatum is modulated by dopamine (9), and related to anhedonia, which is one of core MDD symptoms (10). The most consistent finds from reward system in MDD patients is hypo-activation of striatal regions during the whole reward processing, such as salience, anticipation, feedback, and learning (11). Neuroimaging studies also investigated the neural predictors of treatment outcome and found reward-related function is predictive of treatment outcome. Previous research focused on pretreatment prognostic measures of treatment response to antidepressant agents. The most prominent findings showed that higher anterior cingulate activity before treatment was predictive of positive response to treatment of MDD (12, 13). A meta-analysis study indicates that increased pretreatment rostral anterior cingulate cortex activity is a reliable marker of treatment response (14). Nonetheless, there is little research on early biomarkers during the treatment that can be used to predict later response to antidepressant agents. One study revealed that greater reduction of activation to fearful facial expressions across the anterior cingulate, insula, amygdala, and thalamus after 1-week Selective and Serotonin Reuptake Inhibitors (SSRI) treatment was associated with 6-week clinical response for MDD patients (15).

We mainly focused on 2-week neural changes of the striatum-based circuits since the deficits of these circuits have been constantly linked to MDD (16, 17). As a key node of the reward circuit, striatum has extensive interconnections with the prefrontal cortex, and engages in complex interactions among dopaminergic, serotoninergic, and glutamatergic systems in function (18), making it a critical therapeutic target of antidepressants. Previous studies have shown that striatal activation and cortico-striatal functional connectivity (FC) during reward processing could be altered after different antidepressant treatments (19, 20). Besides, pre-treatment striatal activity and FC is associated with clinical symptom improvement after treatment (21, 22). The striatum has been proposed to functionally divide into several subregions, supporting affective, cognitive, and motor processing, respectively (23), and MDD patients often experience difficulties in these domains (24). As one of core MDD symptoms, anhedonia may result from deficits of the brain's reward system (25, 26), which were associated with abnormality of the striatum-based circuits (17, 25, 27). Psychomotor retardation, as a classic clinical symptom of MDD (28), was associated with decrease in the fronto-striatal connectivity and decrease in striatal volume (29, 30). These findings highlight that the striatal circuits may be key to prognosis and treatment response of MDD.

Abnormal communication among functional brain networks has been found in MDD, mainly focused on the default mode network (DMN) related to the rumination, frontoparietal network (FPN) related to cognitive regulation, and somatosensory network (SMN) referring to psychomotor symptoms of MDD (31), suggesting that investigating MDD from the perspective of unified networks of neural dysfunction may yield an integrative framework to understand this disorder. Based on previous researches, we hypothesized that the change of communication of striatum with DMN, FPN, and SMN at early stage of treatment would decide the enduring clinical symptom improvement of MDD patients.

In the current study, we investigated the resting-state FC changes of striatum within 2-week treatment as an imaging biomarker of clinical symptom improvement after 8-week treatment, given that the dopamine neurotransmission in the striatum and its function were impaired in MDD, but could possibly be changed by the treatment (32, 33). We administered duloxetine to first-episode drug-naïve MDD patients, since duloxetine is a dual-action antidepressant, which increases norepinephrine (NE) and serotonin (5-HT).

## Materials and Methods

### Subjects

Thirty-four first-episode drug-naive MDD patients (18 females; mean age was 30 years old) were recruited to undergo clinical symptom assessment at baseline, after 2 and 8 weeks of treatment with duloxetine. They also underwent resting-state fMRI scanning at baseline and after 2 weeks of the treatment. These patients were diagnosed by qualified psychiatrists using the Mini International Neuropsychiatric Interview (MINI) (34), a short structured interview developed to derive diagnoses according to the Diagnostic and Statistical Manual of Mental Disorders, 4th edition (DSM-IV). Inclusion criteria included an acute depressive episode, total score on the 24-item Hamilton Rating Scale for Depression (HRSD_24_) (35) ≥ 20, and illness duration ≤ 24 months. MDD patients with comorbid Axis I disorders, Axis II personality disorders, or intellectual disability were excluded. Other exclusion criteria consisted of serious medical or neurological illness, a history of significant head trauma, substance dependence or abuse within the last year, current or previous use of psychotropic drugs, a history of electroconvulsive therapy (ECT), acutely suicidal or homicidal, current pregnancy or breastfeeding, or any contraindications to an MRI scan.

Among the initially recruited 34 patients at baseline, eight withdrew the informed consent due to not local residents and not convenient to come back for follow-up, and seven discontinued medication due to the side effect or some personal reasons. A total of 19 patients who finished all MRI scanning and clinical assessment were finally included in this study (see **Figure S1**). This study was carried out in accordance with the recommendation of the Ethics Committee of the Sixth Hospital (Institute of Mental Health) of Peking University with written informed consent from all subjects. All subjects gave written informed consent in accordance with the Declaration of Helsinki. The protocol was approved by the Ethics Committee of the Sixth Hospital (Institute of Mental Health) of Peking University.

### Antidepressant Treatment and Clinical Assessment

All the 19 patients included in the present study received duloxetine treatment for 8 weeks, without taking any other psychotropic medication or psychotherapies during this period. They were treated initially with oral duloxetine at a dose of 30–60 mg/day; then the dose was increased to 60–90 mg/day within the first two weeks, and continued at this level until they finished the 8-week study. The final dose was 60 mg/day for 17 patients, and 90 mg/day for 2 patients. The dose adjustment was accorded to the clinical judgment of the psychiatrist and the patient's consent and findings in our study are not driven by dose effects. (see **Table S2**). Clinical ratings were administered at baseline, the end of 2nd week, and the end of 8th week, using HRSD24.

### fMRI Data Acquisition

Brain imaging was performed on a 3.0 T scanner (Siemens Magnetom Trio; Siemens Medical Solutions, Erlangen, Germany) in the 306th hospital of People's Liberation Army of China. For resting-state scanning, subjects were instructed to keep their eyes closed, to remain still without head movement, and not to fall asleep or think of anything in particular. The resting-state functional images were collected with a gradient-echo echo-planar imaging sequence with the following parameters: repetition time (TR), 2,000 msec; echo time (TE), 30 msec; flip angle, 90°; matrix, 64×64; field of view, 210×210 mm^2^; slice thickness/gap, 4.0 mm/0.8 mm; and 30 axial slices covering the whole brain. 210 functional volumes were acquired in 7 min. After the functional MRI scanning, high-spatial-resolution structural images were acquired for each subject with the T1-weighted magnetization-prepared rapidly acquired gradient-echo (MPRAGE) sequence (TR, 2,300 msec; TE, 3.01 msec; matrix, 256×256; spatial resolution, 1×1×1 mm^3^; flip angle, 9°; thickness, 1 mm; 176 sagittal slices) to achieve better normalization.

### Image Data Preprocessing

The fMRI images were preprocessed with the Data Processing Assistant for Resting-State fMRI (DPARSF, http://rfmri.org/DPARSF) (36), which is based on SPM12 (http://www.fil.ion.ucl.ac.uk/spm). After removing the first 10 volumes, the remaining 200 volumes were corrected for different slice acquisition timing and head motion. Then, the nuisance signals were regressed out, including signal associated with 24 Friston head-motion parameters, signal from the cerebrospinal fluid and white matter, global signal, and linear signal trend. Derived images were co-registered with the corresponding structural images which were segmented and normalized to the Montreal Neurological Institute (MNI) space using the DARTEL. The functional images were then normalized to the MNI space with the warped parameters, and resampled to 3 × 3 × 3 mm cubic voxels. The transformed images were then band-pass filtered (0.01–0.1 Hz) and spatially smoothed with a full width at half maximum of 6 mm. Subjects with large head displacement (max > 3mm) or head rotation (max > 3 degree) were excluded for further analyses. None of the patients were excluded due to large head motion. Since it has been shown that head motion might result in artefactual inter-individual difference in resting-state metrics (37, 38), we adopted a more strict setting of data preprocessing to control the effect of head motion. In brief, beyond regressing out the nuisance signals associated with 24 Friston head-motion parameters, we further removed or “scrubbed’ bad time points with large head motion. The bad time points were defined as those whose frame-wise displacement (FD) was larger than 0.2 mm (see **Supplementary Material**). On average, it scrubbed 43.89 ± 36.89 frames of functional images for both baseline and 2-week scans (see **Supplementary Material**).

### Functional Connectivity Analysis

The striatum is proposed to divide into 12 representative subregions, which are defined bilaterally in the dorsal caudate (DC) (x = ± 13, y = 15, z = 9), superior ventral striatum (VSs) (x = ± 10, y = 15, z = 0), inferior ventral striatum (VSi)/nucleus accumbens (x = ± 9, y = 9, z = -8), dorsal rostral putamen (DRP) (x = ± 25, y = 8, z = 6), dorsal caudal putamen (DCP) (x = ± 28, y = 1, z = 3), and ventral rostral putamen (VRP) (x = ± 20, y = 12, z = -3), as reported in a study by Di Martino et al. (23). These regions are proposed to be differentially involved in executive control (DC, VSs, and VRP), motor (DRP and DCP), and affective processing (VSs and VSi) (23).

We used a seed-based approach to compute FC of the 12 subregions of the striatum. These subregions were regarded as regions of interest (ROI), and a sphere with 4 mm radius was created for each ROI that covered 7 voxels. For FC computation, the mean BOLD time series for each ROI was extracted, and correlated with the BOLD time series of each voxel within the brain using Pearson's Correlation. Then the derived correlation coefficient was transformed to Fisher's Z score for statistical analyses. For each subject, 12 whole-brain voxel-wise striatal FC maps were created.

### Statistical Analysis

FC alterations (ΔFC) from baseline to 2-week after treatment were calculated for each subject and for each ROI: ΔFC = FC_2W_ – FC_baseline_. The treatment efficacy was evaluated using rate of reduction in HRSD scores: ΔHRSD = (HRSD_baseline_ – HRSD_8W_)/HRSD_baseline_ × 100%. Correlation analyses between the ΔFC and ΔHRSD were subsequently performed across the brain, with age, gender, and education level as covariates. We combined Bonferroni correction and Gaussian random field (GRF) theory for multiple comparison correction. Bonferroni correction corrected for multiple comparisons for the 12 ROIs, resulting in p < 0.0042 (i.e. equal to 0.05/12). GRF theory corrected for multiple comparisons across voxels of the brain. Here we performed two-tailed GRF correction, with voxel p < 0.001 and cluster p < 0.0042 (i.e., voxel p < 0.0005 and cluster p < 0.0021 for each tail). To exclude the possibility that the observed association was driven by any outlier, we also performed robust regression test which could decrease the effect of outlier on the association between two variables. The robust regression tests were performed on extracted ROI mean values, using the function of “robustfit” in Matlab. Furthermore, in order to provide evidence that our results were not an artefact caused by head motion, the functional connectivity analysis and the statistical analysis were rerun with the scrubbed data.

## Results

### Sample Characteristics

The HRSD_24_ scores decreased significantly in MDD patients after treatment over 2 and 8 weeks (all ps < 0.001, **Table 1**). For the 19 MDD patients who completed the whole 8-week treatment and assessment, all but two of them showed a clinical response to duloxetine treatment at the end of 8th week, defined as 50% or more reduction of the HRSD_24_ score from baseline; and 15 of them achieved clinical remission with a HRSD_24_ score of 8 or below.

**Table 1:** Demographic and clinical information.

Demography		Timing	Total HRSD_24_
Gender (M/F)	10/9	Baseline	30.11 ± 4.64
Age (years)	29.68 ± 6.29	2nd week	16.89 ± 5.46
Education (years)	15.95 ± 1.65	8th week	6.53 ± 4.02

Values shown are mean ± SD unless otherwise indicated.

### Positive Correlation of 2-Week ΔFC With Symptom Improvement

The present results revealed that greater ΔFC of striatal subdivisions with brain regions in the FPN and DMN after 2-week treatment was associated with better clinical improvement at 8-week.

**Figure 1:**
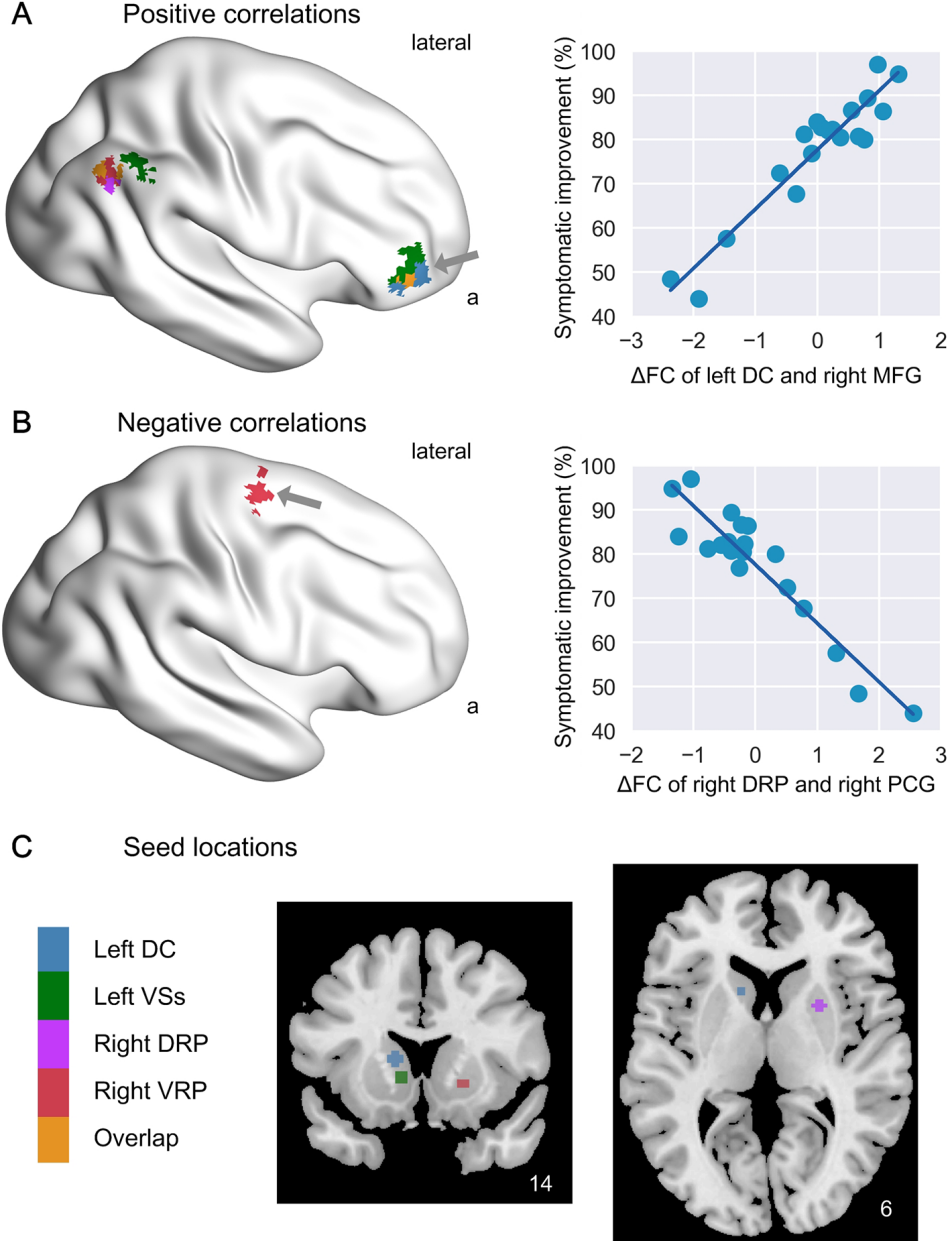
Association of 2-week ΔFC of striatum with 8-week symptom improvement. The image shows the brain regions whose 2-week functional connectivity alterations with different striatal subdivisions (in color) were positively **(A)** and negatively **(B)** correlated with 8-week symptom improvement, and shows the seed locations of the striatal subdivisions **(C)**. On the right are scatters showing examples of positive association (ΔFC of left DC and right MFG, indicated by a grey arrow) and negative association (ΔFC of right DRP and right PCG). DC, dorsal caudate; VSs, superior ventral striatum; DRP, dorsal rostral putamen; VRP, ventral rostral putamen; ΔFC, functional connectivity alteration.

As shown in **Figures 1A, C** and **Table 2**, the ΔFC of the left DC with right middle frontal gyrus (MFG) in the FPN was positively related to treatment response at 8-week (r = 0.94). The ΔFC of the left VSs with right MFG and right inferior parietal lobule (IPL) in the FPN was positively related to treatment response at 8-week as well (r = 0.91 for right MFG and r = 0.87 for the right IPL). The significant clusters of right MFG for the two caudate subdivisions involved in executive control processing (i.e. left DC and left VSs) were overlapped (in orange intersected by blue and green in **Figure 1**), indicative of consistency of the findings. Moreover, for both the right DRP and right VRP, the ΔFC with right angular gyrus (AG) in DMN was positively related to treatment response at 8-week (r = 0.92 for right DRP – right AG, and r = 0.89 for right VRP – right AG; **Figure 1** and **Table 2**). The significant clusters of the right AG were also overlapped (in orange intersected by purple and red), for the two putamen subdivisions (i.e. right DRP and right VRP). Robust regression test also demonstrated that these associations were not affected by any outlier effect (all p < 0.05, FDR corrected). Moreover, the correlations remained significant when the dose level over initial two weeks was added as another covariate, suggesting the results were not driven by dose effects (see **Supplementary Material**).

**Table 2:** Significant association of 2-week ΔFC with 8-week symptom improvement.

Seed	Correlated regions	Network	Voxels	MNI coordinates			Z	r	Robust test
				x	Y	z			
**L DC**	**ROI2 cognitive**								
	R middle frontal gyrus	FPN	56	42	54	0	5.173	0.94	0.001
**L VSs**	**ROI4 cognitive/affective**								
	R middle frontal gyrus	FPN	48	45	48	0	4.590	0.91	0.010
	R inferior parietal lobule	FPN	44	54	–48	45	4.332	0.87	0.028
**R DRP**	**ROI7 motor**								
	R angular gyrus	DMN	47	54	–60	36	4.754	0.92	<0.001
	R precentral gyrus	SMN	38	24	–12	66	–4.145	–0.93	<0.001
**R VRP**	**ROI11 cognitive**								
	R angular gyrus	DMN	49	54	–63	39	4.500	0.89	0.091

DC, dorsal caudate; VSs, superior ventral striatum; DRP, dorsal rostral putamen; VRP, ventral rostral putamen; L, left; R, right; DMN, default mode network; FPN, frontoparietal network; DAN, dorsal attention network; SMN, somatosensory motor network; VN, visual network.

### Negative Correlation of 2-Week ΔFC With Symptom Improvement

By contrast, greater ΔFC of striatal subdivisions with brain regions in the SMN regions after 2-week treatment was associated with worse clinical improvement at 8-week.

**Figure 2:**
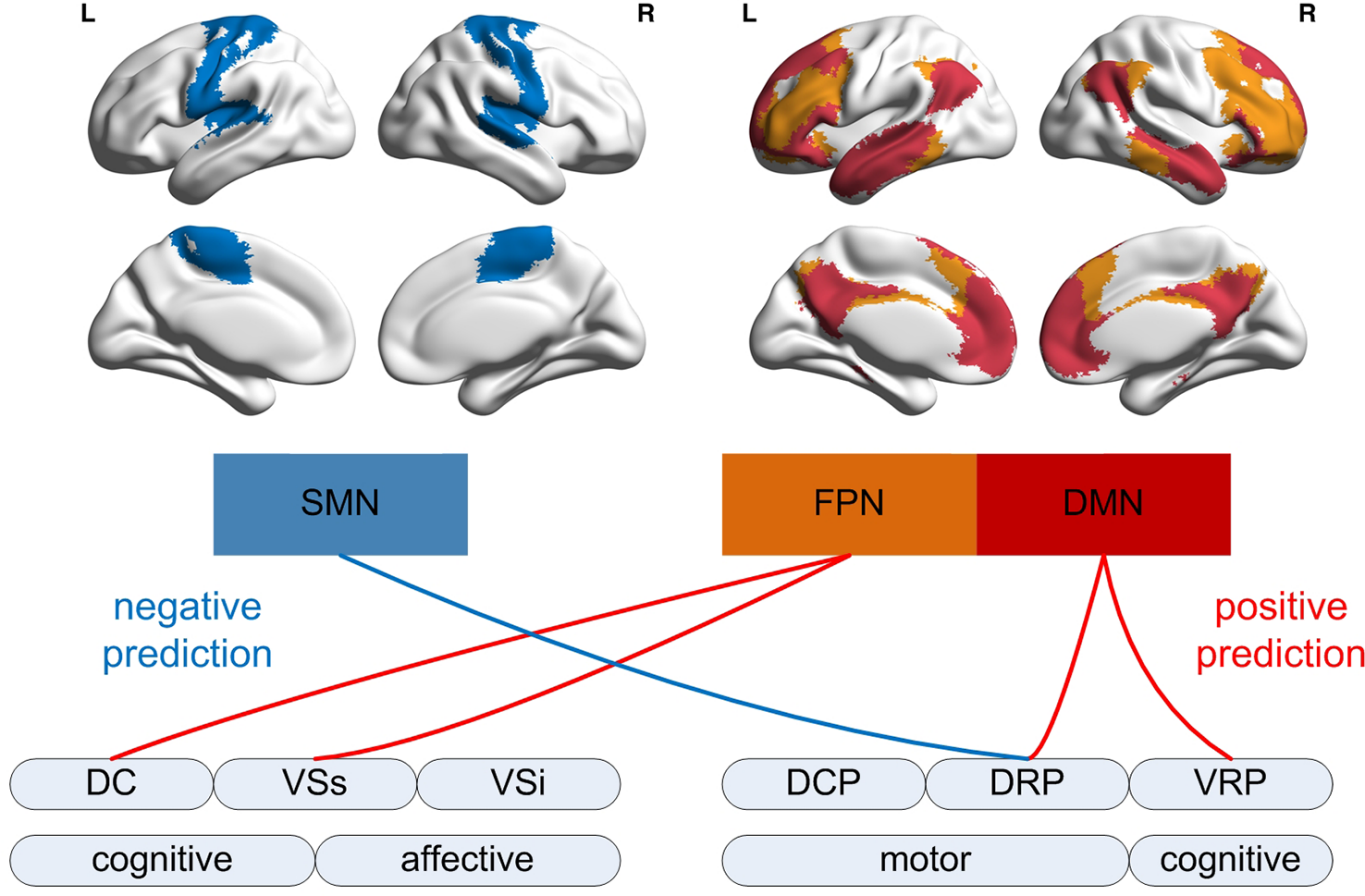
Association of 2-week ΔFC within the network with 8-week symptom improvement. The schematic image shows positive and negative association of functional connectivity changes between different striatal subdivisions with the regions in cortical networks defined by Yeo et al. (40). The positive association mainly involves the striatal subdivision and cortical networks for high-order cognitive processing, while the negative association mainly involves those for visual and motor processing.

As shown in **Figure 1** and **Table 2**, the ΔFC of the right DRP with right precentral gyrus (PrCG) in SMN was negatively related to treatment response at 8-week (r = -0.93). This association of ΔFC and treatment response was not driven by the effect of outlier, as evidenced by robust regression test (p < 0.001). Notably, the negative correlation happened for the cortico-striatal circuit involved in motor processing, while the positive correlation for the circuits involved in high-order cognitive processing (**Figure 2**).

### Validation With Scrubbed Data

In order to further exclude possible confounding effect caused by head motion, we also rerun the data preprocessing with scrubbing which deleted the bad time points with large head motion (frame-wise displacement larger than 0.2 mm). The results of the analyses on scrubbed data almost remained the same as those reported above (**Table S1**), indicating that our findings were not susceptible to artefactual effect induced by head motion.

## Discussion

The cognitive neuropsychological model of drug action proposed by Harmer et al. (39) indicates that the antidepressants affects neural substrates earlier than they change behavior, mood, and social reinforcement in MDD patients. Some research found caudomedial nucleus accumben contains equally high levels of both noradrenaline and dopamine (33), meanwhile duloxetine is a dual-action antidepressant and we suppose it may influence the reward network through increasing norepinephrine of striatum. In addition, the authors further suggest that early neural changes ultimately contribute to later symptomatic improvement (39). Our study was in accord with this model by showing that the early change of the striatum-based circuits after treatment was related to later clinically-observed treatment effects. Interestingly, the positive correlation was observed for the striatal connectivity with the FPN and DMN, which were considered as high-order brain regions involved in cognitive control and emotion processing, while the negative correlation for the striatal connectivity with the SMN considered as low-order primary brain regions (see **Figure 2**).

### Positive Association of ΔFC With Symptom Improvement

The positive association of FC changes with symptom improvement was mainly observed between the left caudate (DC and VSs) and the frontoparietal regions (MFG and IPL), indicating that the more the striatal FC was increased after 2 weeks of treatment with duloxetine, the better the treatment outcome would be after 8 weeks of treatment with duloxetine. The DC and VSs are proposed to be the cognitive subdivision of the striatum, which mainly connects to the cortical regions responsible for cognitive/executive control processing (23). The right MFG and IPL are the core regions of the frontoparietal control network (41), and located within the FPN supporting the cognitive control, defined by Yeo and colleagues (40). A previous study using meta-analysis of resting-state FC has demonstrated aberrant cortico-striatal resting-state FC within the FPN in patients with MDD (31). MDD patients are reliably acknowledged to present significant deficits of executive functions which are related to illness severity and cannot be accounted by motor slowing (42). Poor ability of cognitive control for MDD patients is predictive of their poor response to treatment (43). Our study further indicated that the importance of coordination of the striatum and FPN in action mechanism of the antidepressant. Increased communication between the stratum and FPN after 2-week antidepressant treatment may be beneficial to the cognitive bias and executive function, and then contributes to subsequent mood improvement in daily life (39). Future studies could enroll health controls to further certificate whether normalization of FC of the stratum and FPN after short-term antidepressant treatment can predict the long-term treatment efficacy.

The VSs is also proposed to be the affective/emotion subdivision of the striatum, which is in charge of emotional processing (23). Previous evidence showed decreased activation in the ventral striatum and attenuated FC between ventral striatum with the right MFG during emotion processing in MDD patients (44). The ventral striatum and dorsolateral prefrontal cortex form a neural circuit for affective control and regulation (45), and the deficit of this circuit may contribute to anhedonia, a core feature of MDD (17, 25). Top-down regulation from the PFC has been shown to trigger dopamine release to striatum (46), and dopamine signaling play a key role on mediating reward-related behaviors (47, 48).

Previous researches indeed showed that the abnormally reduced FC of the affective control circuit could be increased by long period of antidepressant treatment, accompanied by clinical symptom improvement (19, 20). Our study further found that the FC changes of the affective control circuit at early stage of treatment, even before the onset of mood change, was positively related to later treatment outcome. MDD patients, especially patients with anhedonia are inclined to positive blockade, which decreases positive emotion experienced during a pleasant event (49). In consistent with cognitive neuropsychological model of antidepressant drug action (39), we speculate that cooperation of affective control circuit may enhance the balance of positive to negative emotional processing, improving the MDD patients' capacity to interpret emotional information in a positive way, which may will be helpful for translating neural changes into clinically symptom improvement in future.

The positive association of FC changes with symptom improvement was also observed between the putamen (right DRP and right VRP) and the DMN regions (AG). The right DRP and right VRP are proposed to be the cognitive and motor subdivision of the putamen, respectively, while the putamen was proposed to be involved in reward-related processing, especially reinforcement learning. Greater cooperation between putamen and AG reflects better mediation of external demands on working memory associated with reward-related reinforcement learning (50). Our study showed antidepressant could change the communication of the putamen and DMN at the early stage of treatment, which may benefit to reward function (51), thus improving subsequent depressive symptoms improvement in a long time.

### Negative Association of ΔFC With Symptom Improvement

Our findings further revealed negative association of FC changes between the striatal motor sub-division (DRP) with the SMN (right PrCG). The DRP in the striatum is involved in various motor-related functions through abundant connections with the motor regions (23), while the PrCG is considered as the primary motor area for movement execution (52). MDD is characterized by psychomotor retardation, which is reflected as slow thought and reduced physical movement (53). Un-medicated MDD patients have been shown to respond slower in simple motor tasks, suggesting deficit in their motor system (24, 54). Previous studies on medicated MDD patients has linked psychomotor retardation to increased activity of the motor-related regions and the striatum (55), but to decrease in the connectivity between these regions (30), which may result from the antidepressant effect. In our study, the negative assoication reflected that decrease in the striatal-SMN FC involved in motor execution after 2-week treatment with duloxetine was related to MDD remission, which is consistent with the finding by Liberg and Rahm (30). We speculated that changing interaction of striatal and SMN at early stage of treatment was important for enduring symptomatic improvement. Future studies are needed to clarify the abnormality of motor-related circuits associated with MDD.

### Implication for Clinical Practice

The current findings provide empirical evidence supporting potential clinical practices that antidepressant aiming to potentiate 5-HT and NE transmission can change the striatal-based circuits at an early stage, and striatal FC changes within 2-week treatment may be an indicator of clinical improvement in long term. MDD patients with greater level of cognition and execution function can achieve better clinical improvement at 8-week. Without inclusion of a control group, the present study could not determine whether 2-week treatment exerted such normalized effects. It will also be necessary for future studies to have a placebo control group or a different MDD group taking other treatment regimen, to clarify the specific drug-induced mechanism underlying predictive effects of early striatum-based FC changes on later clinical improvement.

One limitation of this study was the relatively high rate of drop out of patients (15/34), which is unavoidable in clinical trial. We cannot generalize our findings to the drop-out patients, who probably do not benefit from the treatment. Another related limitation is the relatively small sample size for this study. Future studies are needed to verify the results with a larger sample size. In addition, future studies could assess whether the early neural changes can be used to predict response at an individual in a larger cohort of patients.

In conclusion, the current study revealed that neural changes of the striatal circuits after 2-week antidepressant treatment was associated with later treatment outcome for patients with MDD. The positive association was manifested for functional connectivity between multiple brain networks, especially the FPN and DMN involved in cognitive and affective control, while the negative association mainly focused on the SMN concerned with motor processing.

## Data Availability Statement

The raw data supporting the conclusions of this article will be made available by the authors, without undue reservation, to any qualified researcher.

## Ethics Statement

This study was carried out in accordance with the recommendation of the Ethics Committee of the Sixth Hospital (Institute of Mental Health) of Peking University with written informed consent from all subjects. All subjects gave written informed consent in accordance with the Declaration of Helsinki. The protocol was approved by the Ethics Committee of the Sixth Hospital (Institute of Mental Health) of Peking University. Written informed consent for participation was not required for this study in accordance with the national legislation and the institutional requirements.

## Author Contributions

JA and LL wrote the paper. LW, Y-AS, and YW enrolled the patients. KL and YZ carried out the MRI scans. QK gave suggestions on the paper. TS and CY designed the study.

## Funding

We are thankful for the funding from the National Natural Science Foundation of China (No. 81630031; 81671774), Beijing Municipal Science and Technology Project (Z171100000117016), the Capital Foundation of Medicine Research and Development (2016-1-4111), National Key R&D Program of China (2017YFC1309902), and the Hundred Talents Program of the Chinese Academy of Sciences.

## Conflict of Interest

The authors declare that the research was conducted in the absence of any commercial or financial relationships that could be construed as a potential conflict of interest.
