# Supplementary material for: Striatal Functional Connectivity Alterations After Two-Week Antidepressant Treatment Associated to Enduring Clinical Improvement in Major Depressive Disorder
Source: Front Psychiatry. 2019 Dec 6;10:884. doi: 10.3389/fpsyt.2019.00884 (PMC6915079; doi:10.3389/fpsyt.2019.00884)
Supplement: Supplementary file 1 [file Presentation_1.pdf]

Supplementary Material

Data analyses with scrubbed data

Since head motion may cause artefactual inter-individual difference in resting-state metrics (Power, et al., 2012), we adopted a more strict setting of data preprocessing to exclude possible confounding effect caused by head motion, and then reanalyzed the data to verify our main results. Specifically, beyond regressing out the nuisance signals associated with 24 Friston head-motion parameters, we further removed or "scrubbed' bad time points with large head motion before computing FC (Power, et al., 2014). The bad time points were defined as those with head motion larger than 0.2 mm frame-wise displacement (FD). All other data preprocessing settings remained unchanged. The striatal FC and the FCA from baseline to 2-week after treatment were calculated, and the striatal FCA was correlated with 8-week symptomatic improvement (i.e. ΔHRSD from baseline to 8-week), with age, gender, and education level as covariates.

The analyses with scrubbed data verified the results reported in the main text (Table S1). In detail, greater FCA of the left caudate (DC and VSs) with right middle frontal gyrus and right inferior parietal lobule in the FPN was associated with better clinical improvement at 8-week. FCA of the right putamen (DRP and VRP) with right angular gyrus in the DMN was positively related to treatment response at 8-week. A negative predictive effect of FCA on clinical improvement at 8-week was found between the right putamen (DRP) with right precentral gyrus in the SMN. The analysis with the scrubbed data additionally revealed a positive predictive effect of FCA between the right DCP and left VRP on clinical improvement at 8-week.

Power, J.D., Barnes, K.A., Snyder, A.Z., et al. (2012) Spurious but systematic correlations in functional connectivity MRI networks arise from subject motion. Neuroimage, 59:2142-54.

Power, J.D., Mitra, A., Laumann, T.O., et al. (2014) Methods to detect, characterize, and remove motion artifact in resting state fMRI. Neuroimage, 84:320-41.

Table S1 Significant predictive effect of 2-week FCA on 8-week symptom improvement using scrubbed data

Seed	Correlated regions	Network	Voxels	MNI coordinates			Z	r	Robust test
				x	y	z			
**L DC**	**ROI2 cognitive**								
	R middle frontal gyrus	FPN	48	42	54	0	5.267	0.92	<0.001
**L VSs**	**ROI4 cognitive/affective**								
	R middle frontal gyrus	FPN	30	42	45	0	4.575	0.89	0.011
	R inferior parietal lobule	FPN	31	54	-48	45	4.001	0.86	0.025
**R DRP**	**ROI7 motor**								
	R angular gyrus	DMN	36	54	-60	36	4.434	0.90	<0.001
	R precentral gyrus	SMN	30	24	-12	66	-4.114	-0.93	<0.001
**R DCP**	**ROI9 motor**								
	L VRP	NA	38	-18	9	0	4.675	0.95	<0.001
**R VRP**	**ROI11 cognitive**								
	R angular gyrus	DMN	28	51	-57	54	4.168	0.87	0.047

DC, dorsal caudate; VSs, superior ventral striatum; DRP, dorsal rostral putamen; DCP, dorsal caudal putamen; VRP, ventral rostral putamen; L, left; R, right; DMN, default mode network; FPN, frontoparietal network; DAN, dorsal attention network; SMN, somatosensory motor network; VN, visual network, NA, not applicable.

***, $p < 0.001$; **, $p < 0.01$; *, $p < 0.05$.

Data analyses with Drug Dose

Among the 19 MDD patients, the final dose was 60 mg/day for 17 patients, and 90 mg/day for 2 patients. For the 2 patients with final dose of 90 mg/day, we did some analysis to investigate whether the drug dose have effect on the result. We found their ΔFC was not significantly higher than those with a lower dose (see the table S2).

Furthermore, adding dose as a covariate did not alter the significant level of the correlation analyses between ΔFC and ΔHRSD (all $p < 0.0001$). Therefore, the findings in our study are not driven by dose effects. “Moreover, the correlations remained significant when the dose level over initial two weeks was added as another covariate, suggesting the results were not driven by dose effects.”

Table S2 ΔFC for the two subjects at a higher dose compared to others

Seed	Correlated region	Sub 07	Sub 09	Others
**L DC**	R middle frontal gyrus	1.31	-0.211	-0.065
**L VSs**	R middle frontal gyrus	1.145	0.034	-0.069
	R inferior parietal lobule	1.395	-0.197	-0.07
**R DRP**	R angular gyrus	1.111	0.327	-0.085
	R precentral gyrus	-1.348	-0.766	0.124
**R VRP**	R angular gyrus	1.175	0.945	-0.125

The table shows the ΔFC (in z-score) after controlling for the effect of age, gender, and education level. Sub07,09 took the medication at a higher dose.

Figure S1 flow chart

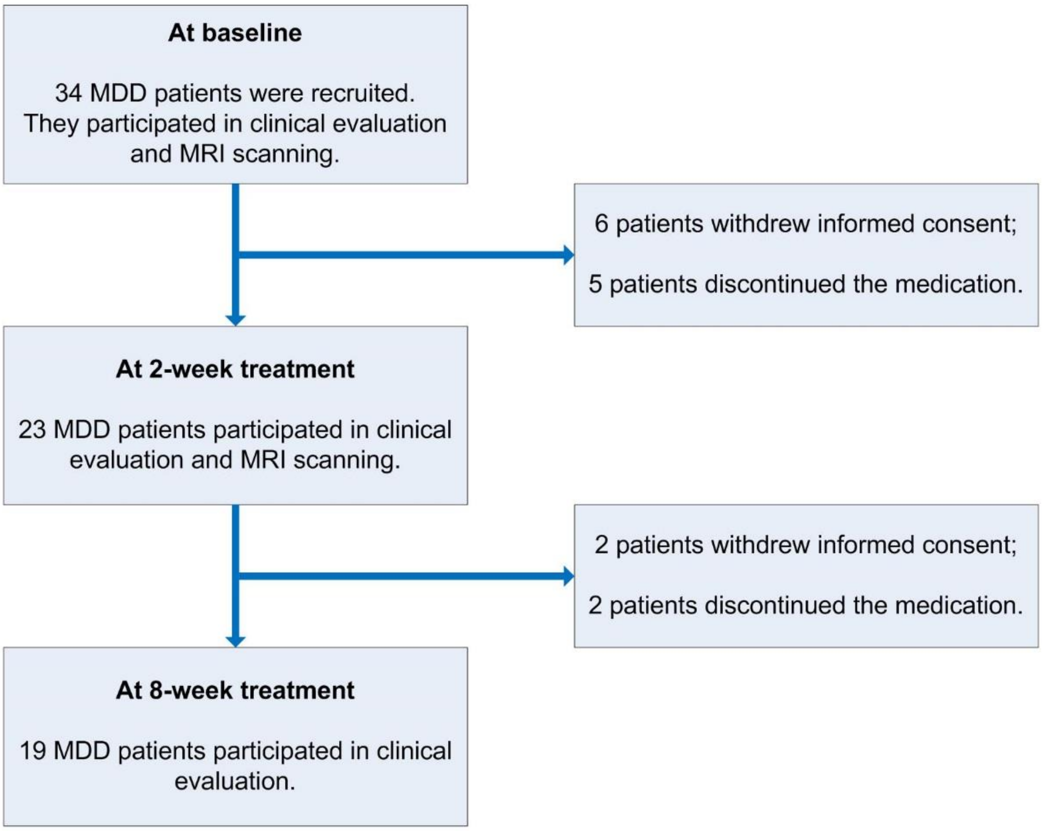

Figure S1: Among the initially recruited 34 patients at baseline, eight withdrew the informed consent due to not local residents and not convenient to come back for follow-up, and seven discontinued medication due to the side effect or some personal reasons. A total of 19 patients finished all MRI scanning and clinical assessment.
